# Supplementary material for: JAK2 inhibition mediates clonal selection of RAS pathway mutations in myeloproliferative neoplasms
Source: Nat Commun. 2025 Jul 8;16:6270. doi: 10.1038/s41467-025-60884-1 (PMC12234676; doi:10.1038/s41467-025-60884-1)
Supplement: Supplementary file 1 — Supplementary Information [file 41467_2025_60884_MOESM1_ESM.pdf]

JAK2 inhibition mediates clonal selection of RAS pathway mutations in myeloproliferative neoplasms

Inventory of Supplementary Information

Supplementary figures

Figure S1
Figure S2
Figure S3
Figure S4
Figure S5
Figure S6
Figure S7
Figure S8

Supplementary tables

Table S1
Table S2
Table S3
Table S4
Table S5
Table S6
Table S7
Table S8
Table S9
Table S10
Table S11
Table S12

Figure S1

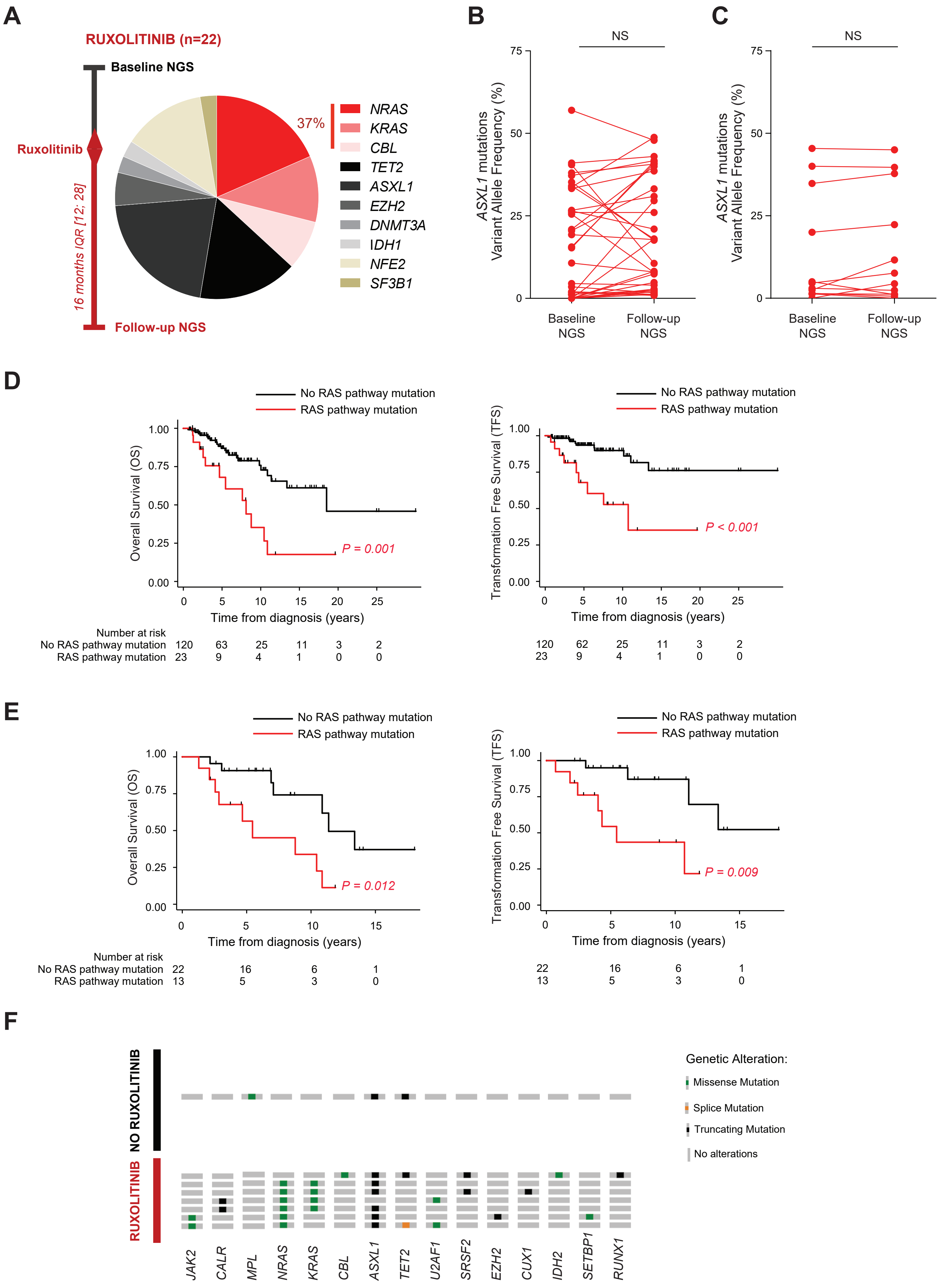
A
RUXOLITINIB (n=22)
Baseline NGS
Ruxolitinib
16 months IQR [12; 28]
Follow-up NGS
37%
NRAS
KRAS
CBL
TET2
ASXL1
EZH2
DNMT3A
IDH1
NFE2
SF3B1
B
NS
ASXL1 mutations
Variant Allele Frequency (%)
75
50
25
0
Baseline NGS
Follow-up NGS
C
NS
ASXL1 mutations
Variant Allele Frequency (%)
75
50
25
0
Baseline NGS
Follow-up NGS
D
No RAS pathway mutation
RAS pathway mutation
Overall Survival (OS)
1.00
0.75
0.50
0.25
0.00
P = 0.001
0 5 10 15 20 25
Time from diagnosis (years)
Number at risk
No RAS pathway mutation 120 63 25 11 3 2
RAS pathway mutation 23 9 4 1 0 0
No RAS pathway mutation
RAS pathway mutation
Transformation Free Survival (TFS)
1.00
0.75
0.50
0.25
0.00
P < 0.001
0 5 10 15 20 25
Time from diagnosis (years)
120 62 25 11 3 2
23 9 4 1 0 0
E
No RAS pathway mutation
RAS pathway mutation
Overall Survival (OS)
1.00
0.75
0.50
0.25
0.00
P = 0.012
0 5 10 15
Time from diagnosis (years)
Number at risk
No RAS pathway mutation 22 16 6 1
RAS pathway mutation 13 5 3 0
No RAS pathway mutation
RAS pathway mutation
Transformation Free Survival (TFS)
1.00
0.75
0.50
0.25
0.00
P = 0.009
0 5 10 15
Time from diagnosis (years)
22 16 6 1
13 5 3 0
F
NO RUXOLITINIB
RUXOLITINIB
JAK2
CALR
MPL
NRAS
KRAS
CBL
ASXL1
TET2
U2AF1
SRSF2
EZH2
CUX1
IDH2
SETBP1
RUNX1
Genetic Alteration:
Missense Mutation
Splice Mutation
Truncating Mutation
No alterations

Figure S1. Acquired mutations after ruxolitinib exposure and their clinical impact. (A) Pie Chart depicting the additional mutations acquired after ruxolitinib treatment in 22 patients with myelofibrosis for which molecular evaluation was available prior to and after ruxolitinib initiation. **(B-C)** *ASXL1* mutations variant allele frequency longitudinal evolution in patients with myelofibrosis exposed (n=45 patients) **(B)** or not exposed (n=28 patients) **(C)** to ruxolitinib between baseline and follow-up molecular evaluation. Variant allele frequency was considered zero when mutations were absent at baseline or follow-up molecular evaluation. Statistical significance was determined using Mann-Whitney test in comparison to baseline molecular evaluation. **(D-E)** Kaplan-Meier curves of overall survival (left) or transformation-free survival (right), according to presence of RAS pathway mutations, in the whole study cohort **(D)** or in the DIPSS intermediate-2/high ruxolitinib-treated patients only **(E)**. COX regression analysis was used for comparing the groups. **(F)** Oncoprints showing the mutational landscape of AML/MDS transformations among RAS mutated patients, treated or not with ruxolitinib. Data was available for n=7/8 ruxolitinib treated patients and n=1/1 non-ruxolitinib treated patient. Data provided as Source Data file.

Figure S2

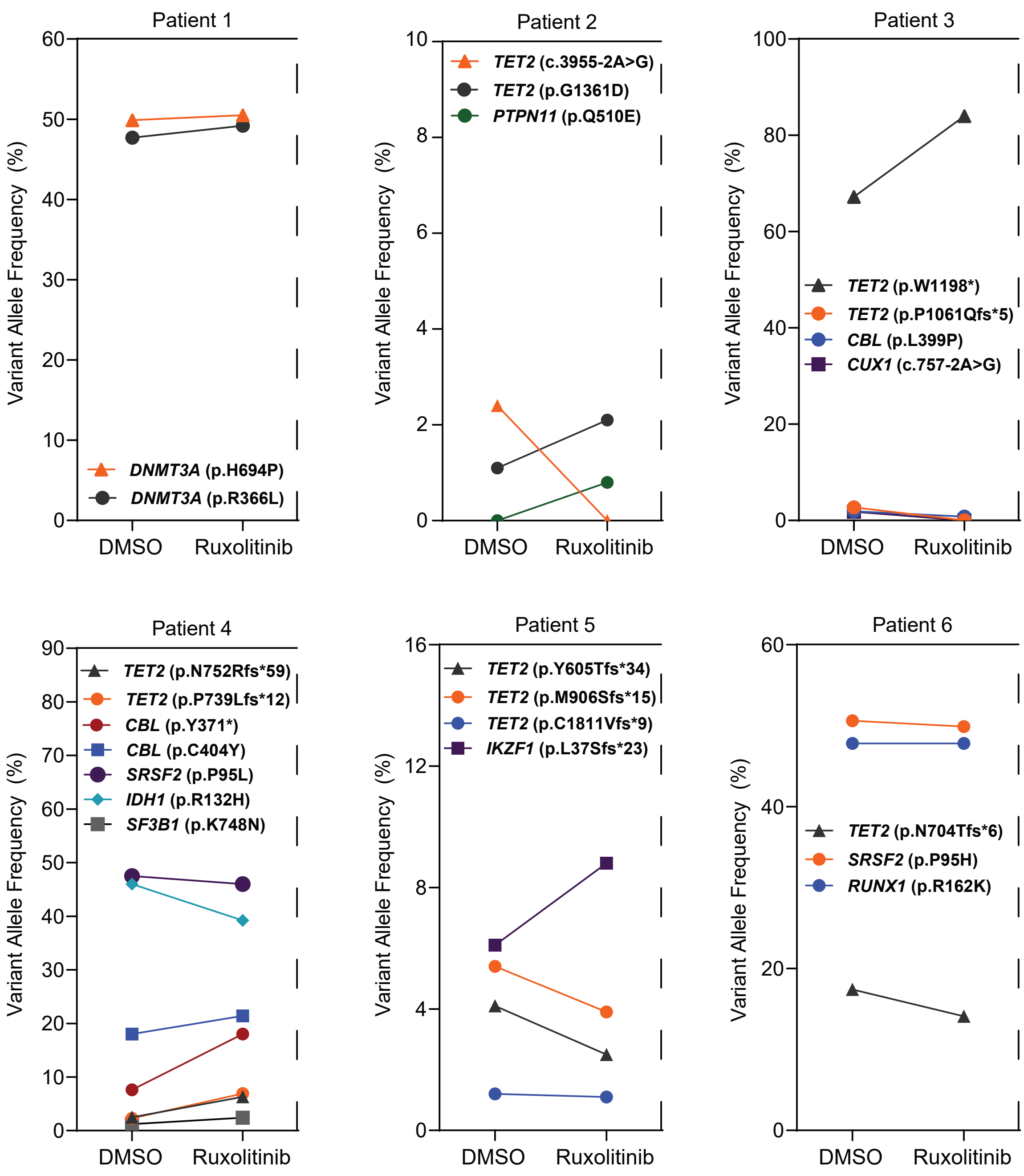

Figure S2. Non-RAS mutant clones evolution under ruxolitinib treatment. Graphical representation of the allele burden of all mutations detected 10 days after DMSO or ruxolitinib (20 nM) *in vitro* treatment of $CD34^+$ hematopoietic cells derived from n=6 MPN patients harboring RAS mutations. Data provided as Source Data file.

Figure S3

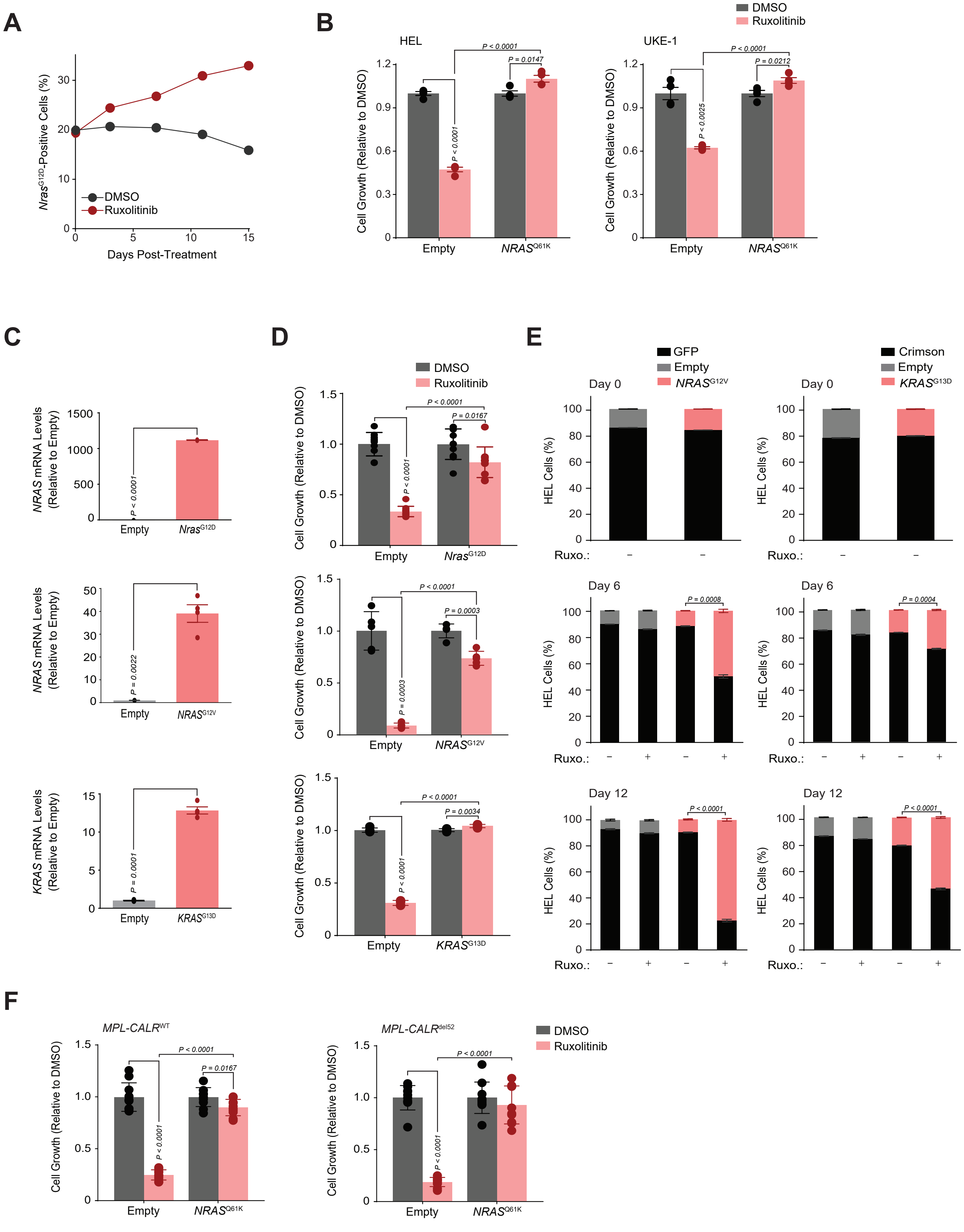
A
B
C
D
E
F
$Nras^{G12D}$-Positive Cells (%)
Days Post-Treatment
DMSO
Ruxolitinib
HEL
UKE-1
Cell Growth (Relative to DMSO)
Empty
$NRAS^{Q61K}$
$NRAS$ mRNA Levels (Relative to Empty)
$KRAS$ mRNA Levels (Relative to Empty)
$Nras^{G12D}$
$NRAS^{G12V}$
$KRAS^{G13D}$
GFP
Crimson
Day 0
Day 6
Day 12
HEL Cells (%)
Ruxo.:
MPL-$CALR^{WT}$
MPL-$CALR^{del52}$

Figure S3. *RAS* mutations confer a fitness advantage upon ruxolitinib exposure. (A) CD45.1/2*Nras*^{G12D} lineage negative murine bone marrow cells were mixed with CD45.1 *Nras*^{WT} lineage negative murine bone marrow cells at a 20/80 cellular ratio, prior to treatment with DMSO or ruxolitinib (0.25 µM) for up to 15 days. Percentage of CD45.1/2*Nras*^{G12D} cells is represented at the indicated time points. Mean of n=2 biological replicates. **(B)** Growth inhibition of indicated human AML cell lines expressing either an Empty or a *NRAS*^{Q61K} encoding vector after 6 days of ruxolitinib treatment (3 µM and 15 µM for HEL and UKE-1 respectively). Statistical significance determined using two-tailed Welch's t-test. Error bars represent mean of n=4 biological replicates ± SEM. **(C)** qRT-PCR for *NRAS* or *KRAS* expression level in HEL cell line infected with an Empty, a *Nras*^{G12D}, a *NRAS*^{G12V} or a *KRAS*^{G13D} encoding vector. Statistical significance determined using two-tailed Welch's t-test. Error bars represent mean of n=4 biological replicates ± SEM. **(D)** Growth inhibition of indicated HEL human AML cell line expressing either an Empty or a *Nras*^{G12D}, a *NRAS*^{G12V} or a *KRAS*^{G13D} encoding vector after 6 days of ruxolitinib treatment (3 µM). Statistical significance determined using two-tailed Welch's t-test. Error bars represent mean of n=5 (*NRAS*^{G12V} and *KRAS*^{G13D}) or n=10 (*Nras*^{G12D}) biological replicates ± SD. **(E)** Percentage of GFP^{+} and GFP^{-}_Empty or GFP^{-}_*NRAS*^{G12V} HEL cells, or Crimson^{+} and Crimson^{-}_Empty or Crimson^{-}_*KRAS*^{G13D} at the indicated days after ruxolitinib (Ruxo.) treatment. Statistical significance determined using two-tailed Welch's t-test. Error bars represent mean of n=3 biological replicates ± SEM. **(F)** Growth inhibition of Ba/F3 *MPL-CALR*^{WT} and Ba/F3 *MPL-CALR*^{del52} cells expressing either an Empty or a *NRAS*^{Q61K} encoding vector after 3 days of ruxolitinib treatment (75nM). Statistical significance determined using two-tailed Welch's t-test. Error bars represent mean of n=10 biological replicates ± SD.
Data provided as Source Data file.

Figure S4

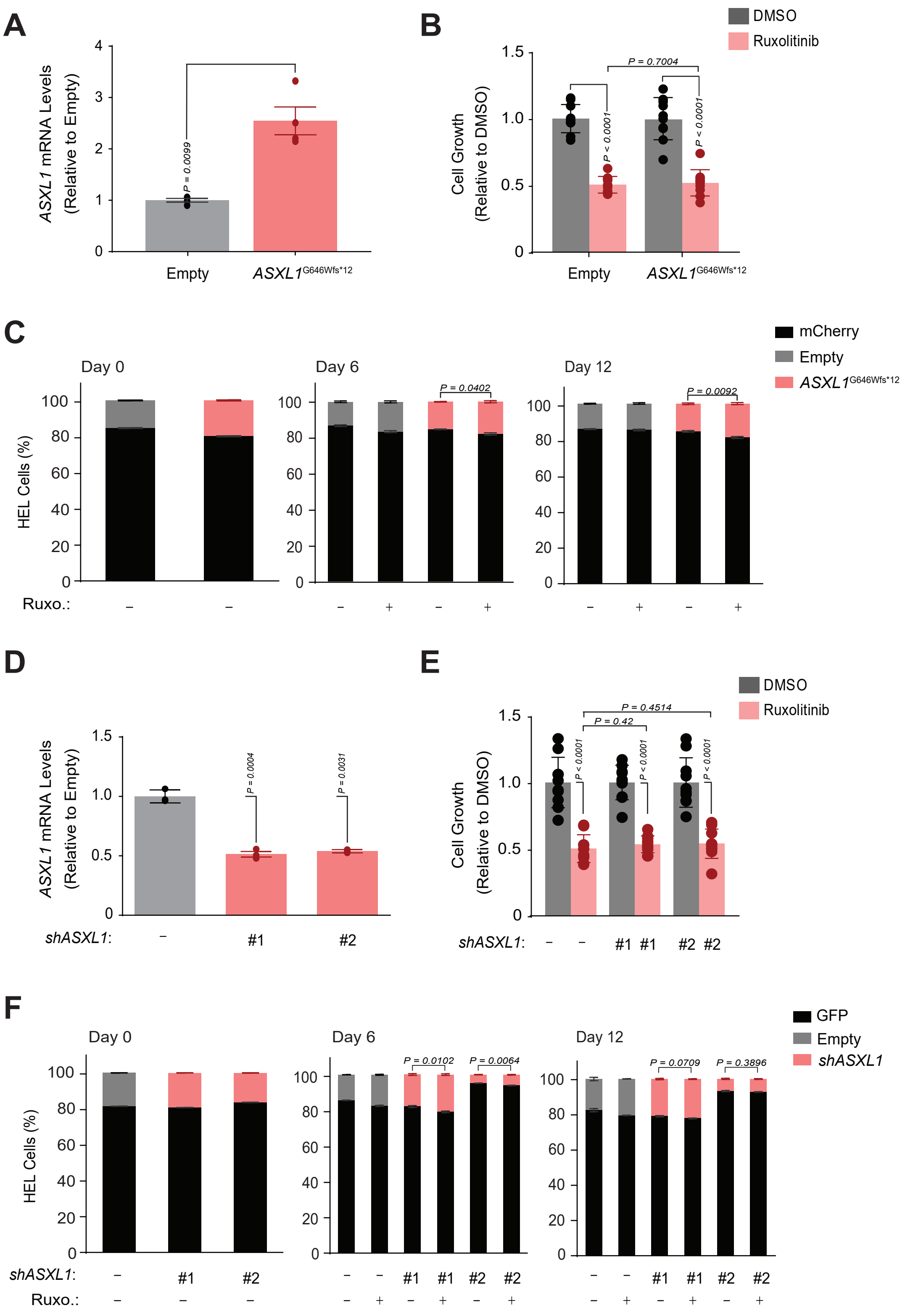

A
B
C
D
E
F
ASXL1 mRNA Levels (Relative to Empty)
P = 0.0099
Empty
ASXL1G646Wfs*12
DMSO
Ruxolitinib
Cell Growth (Relative to DMSO)
P = 0.7004
P < 0.0001
mCherry
Empty
ASXL1G646Wfs*12
Day 0
Day 6
Day 12
HEL Cells (%)
P = 0.0402
P = 0.0092
Ruxo.:
P = 0.0004
P = 0.0031
shASXL1:
P = 0.4514
P = 0.42
GFP
Empty
shASXL1
P = 0.0102
P = 0.0064
P = 0.0709
P = 0.3896

Figure S4. *ASXL1* mutations do not confer a strong fitness advantage upon ruxolitinib exposure. (A) qRT-PCR for *ASXL1* expression level in HEL cell line infected with an Empty or an *ASXL1*^{G646Wfs*12} encoding vector. Statistical significance determined using two-tailed Welch's t-test. Error bars represent mean of n=4 biological replicates ± SEM. **(B)** Growth inhibition of indicated HEL human AML cell line expressing either an Empty or an *ASXL1*^{G646Wfs*12} encoding vector after three days of ruxolitinib treatment (3 µM). Statistical significance determined using two-tailed Welch's t-test. Error bars represent mean of n=10 biological replicates ± SD. **(C)** Percentage of mCherry$^+$ and mCherry$^-$_Empty or mCherry$^-$_*ASXL1*^{G646Wfs*12} HEL cells at the indicated days after ruxolitinib (Ruxo.) treatment. Statistical significance determined using two-tailed Welch's t-test. Error bars represent mean of n=3 biological replicates ± SEM. **(D)** qRT-PCR for *ASXL1* expression level in HEL cell line infected with an Empty or two sh*ASXL1* encoding vectors (#1 and #2). Statistical significance determined using two-tailed Welch's t-test. Error bars represent mean of n=3 biological replicates ± SD. **(E)** Growth inhibition of indicated HEL human AML cell line expressing either an Empty or two sh*ASXL1* encoding vectors (#1 and #2) after 3 days of ruxolitinib treatment (3 µM). Statistical significance determined using two-tailed Welch's t-test. Error bars represent mean of n=10 biological replicates ± SD. **(F)** Percentage of GFP$^+$ and GFP$^-$_Empty or GFP$^-$_sh*ASXL1* (#1 and #2) HEL cells at the indicated days after ruxolitinib (Ruxo.) treatment. Statistical significance determined using two-tailed Welch's t-test. Error bars represent mean of n=3 biological replicates ± SEM.
Data provided as Source Data file.

Figure S5

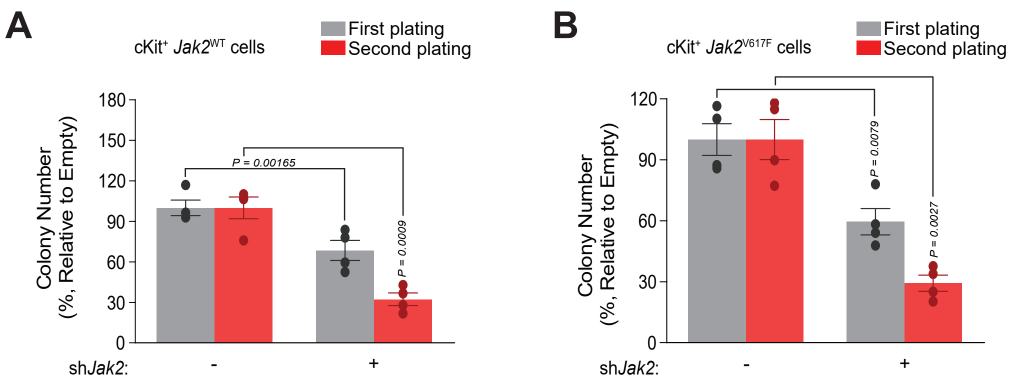

Figure S5. *JAK2* knockdown is associated with decreased colony-forming ability in the absence of RAS mutation. (A-B) Colony formation assay of *Jak2*^{WT} **(A)** or *Jak2*^{V617F} **(B)** C57BL/6 primary bone marrow c-Kit$^+$ murine cells expressing either an Empty, or a sh*Jak2* encoding vector at least 6 days after GFP sorting. Statistical significance determined using two-tailed Welch's t-test. Error bars represent mean of n=4 biological replicates ± SEM.
Data provided as Source Data file.

Figure S6

A

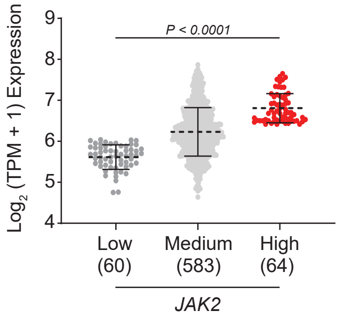

B

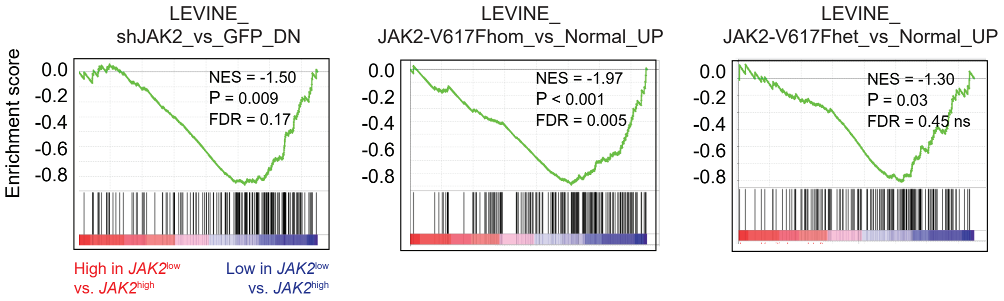

C

#	RAS/MAPK enriched gene sets	NES	p-value	FDR	Size gene set
1	CHIARADONNA_NEOPLASTIC_TRANSFORMATION_KRAS_UP	2.13	0.0E+00	0.0E+00	114
2	MCMURRAY_TP53_HRAS_COOPERATION_RESPONSE_UP	1.81	5.3E-03	9.5E-04	21
3	RAS_NCI_V2	1.76	0.0E+00	1.7E-03	213
4	CHIARADONNA_NEOPLASTIC_TRANSFORMATION_KRAS_CDC25_UP	1.75	1.7E-03	1.9E-03	54
5	SWEET_KRAS_TARGETS_UP	1.71	0.0E+00	2.6E-03	75
6	CASTELLANO_HRAS_AND_NRAS_TARGETS_UP	1.62	1.1E-02	6.2E-03	6
7	SWEET_LUNG_CANCER_KRAS_UP	1.45	1.3E-03	2.8E-02	411
8	HALLMARK_KRAS_SIGNALING_UP	1.44	6.4E-03	3.1E-02	167
9	YOSHIMURA_MAPK8_TARGETS_UP	1.37	0.0E+00	5.2E-02	853
10	GOBP_MAPK_CASCADE	1.23	2.8E-02	1.5E-01	620
11	GOBP_POSITIVE_REGULATION_OF_MAPK_CASCADE	1.22	5.0E-02	1.5E-01	384

D

#	Cell cyle enriched gene sets	NES	p-value	FDR	Size gene se
1	HALLMARK_E2F_TARGETS	2.90	0.0E+00	0.0E+00	195
2	HALLMARK_G2M_CHECKPOINT	2.76	0.0E+00	0.0E+00	187
3	HALLMARK_MYC_TARGETS_V1	2.31	0.0E+00	0.0E+00	193
4	GOBP_MITOTIC_CELL_CYCLE	2.27	0.0E+00	0.0E+00	831
5	GOBP_CELL_CYCLE_DNA_REPLICATION	2.24	0.0E+00	0.0E+00	41
6	GOBP_DNA_STRAND_ELONGATION	2.21	0.0E+00	0.0E+00	36
7	GOBP_MITOTIC_CELL_CYCLE_CHECKPOINT_SIGNALING	2.12	0.0E+00	0.0E+00	129
8	GOBP_MITOTIC_SPINDLE_ELONGATION	2.10	0.0E+00	0.0E+00	12
9	GOBP_CELL_CYCLE_PROCESS	2.09	0.0E+00	0.0E+00	1088
10	GOBP_REGULATION_OF_CELL_CYCLE_PROCESS	2.09	0.0E+00	0.0E+00	632
11	GOBP_CELL_CYCLE_CHECKPOINT_SIGNALING	2.02	0.0E+00	0.0E+00	175
12	HALLMARK_MYC_TARGETS_V2	1.98	0.0E+00	1.5E-04	57
13	HALLMARK_MITOTIC_SPINDLE	1.95	0.0E+00	4.4E-04	196
14	GOBP_MEIOTIC_CELL_CYCLE	1.95	0.0E+00	4.2E-04	213
15	GOBP_CELL_CYCLE	1.92	0.0E+00	4.4E-04	1535
16	GOBP_REGULATION_OF_MEIOTIC_CELL_CYCLE	1.50	1.9E-02	1.9E-02	51

Figure S6. *JAK2* "low" patient group defined within the Beat AML v2 dataset is associated with *JAK2* low expression and *JAK2* gene targets and mutation gene set signatures. (A) Dotplot for *JAK2* expression level (log2 (TPM+1)) according to Beat AML v2 JAK2 "low" (n=60) and "high" (n=64) patient groups definition. Statistical significance determined using one-way ANOVA with Tukey's multiple comparisons test. Error bars represent mean ± SD. **(B)** Genome-wide GSEA for enrichment in expression changes between Beat AML v2 *JAK2* "low" (n=60) versus "high" (n=64) patient groups showing the association with JAK2 gene target and mutated gene set signatures. **(C-D)** P value and NES for gene sets related to RAS/MAPK **(C)** and Cell Cycle **(D)** pathways enriched in the Beat AML v2 *JAK2* "low" patient group. The NES was computed based on the Kolmogorov-Smirnov enrichment test. The statistical significance of the enrichment score for a given gene set was assessed using the nominal p-value from a two-tailed permutation test.

Figure S7

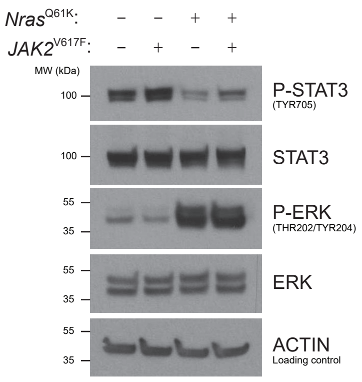

Figure S7. *NRAS*^{Q61K} overexpression is associated with decreased JAK/STAT activation and is partially rescued by JAK2V617F overexpression. Western blot for P-STAT3 (TYR705), STAT3, P-ERK (THR202/TYR204), ERK and ACTIN (loading control), in HEL cell line expressing either Empty, *Nras*^{Q61K}, *JAK2*^{V617F} or *Nras*^{Q61K} and *JAK2*^{V617F} encoding vectors. Experiment was performed at least twice with similar results.

Figure S8

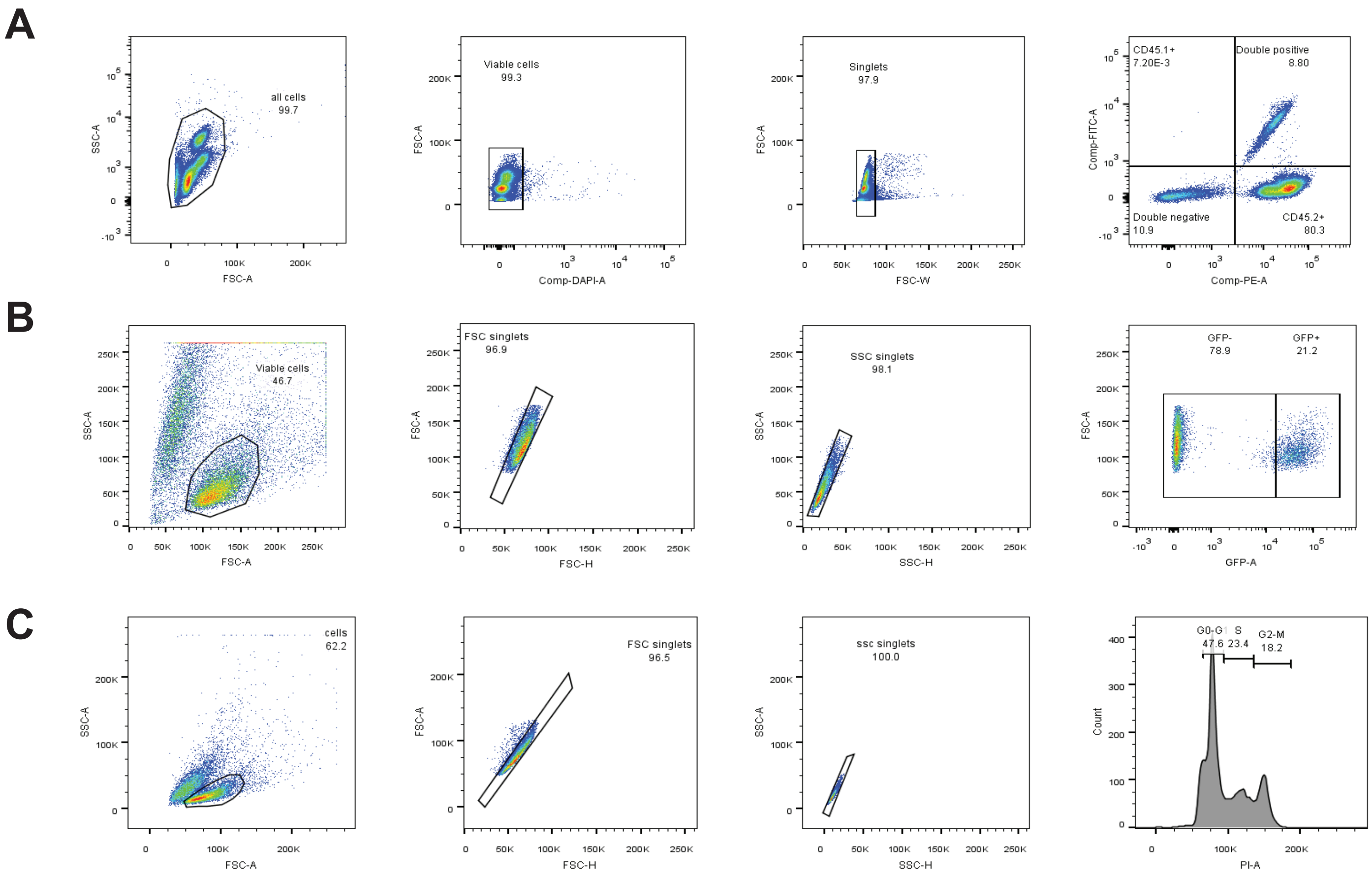

Figure S8. Flow Cytometry Gating Strategy. (A) CD45.1/CD45.2 murine cells in figure panels 4A, 4D, S3A. Total murine cells were gated based on their size and granulosity. Viable cells were isolated by plotting against DAPI. Doublets were excluded from the DAPI-viable cells using FSC-A/FSC-W. Percentage of CD45.1^{+} and CD45.2^{+} competing cells were determined by plotting FITC-A on PE-A. **(B)** Fluorescently labelled cells competition assays in figure panels 4K, 4M, S3E, S4C, S4F. Viable cells were determined based on their size and granulosity (FSC-A/SSC-A). Singlets were identified using FSC-A/FSC-H and SSC-A/SSC-H. Isolated singlets were plotted for fluorescent marker against FSC-A to discriminate the negative cells (empty or RAS mutated) from the positive competing cells. **(C)** Cell cycle analysis in figure panel 6F. Viable cells were isolated according to their size and granulosity (FSC-A/SSC-A). Singlets gated using FSC-A/FSC-H and SSC-H/SSC-A were plotted for PI against count to distribute cells according to their cell cycle phase.

Table S1: Myelofibrosis patients characteristics according to ruxolitinib treatment status. *IQR=Interquartile range, nb= number of patients, PMF=Primary Myelofibrosis, PV=Polycythemia Vera, ET=Essential Thrombocythemia, MDS/MPN=Myelodysplastic Syndrome/Myeloproliferative Neoplasm IPSS= International Prognostic Scoring System, DIPSS= Dynamic International Prognostic Scoring System,HMR= high molecular risk mutations (ASXL1, EZH2, SRSF2, IDH1/2 and/or U2AF1).Continuous variables are reported as medians and interquartile ranges (IQR), while categorical variables are reported as numbers and proportions. Comparison of continuous and categorical variables between subgroups was performed by two-sided Mann-Whitney test and Fisher's exact test, respectively.*

	**All MF patients (n = 143)**	**RUXOLITINIB treated MF patients (n = 72)**	**Non RUXOLITINIB treated MF patients (n = 71)**	**pval**
**Age (years), median (IQR)**	61 (51; 67)	61.5 (52; 69)	60 (49; 66)	0.126
**Female, nb (%)**				0.405
No	74 (51.75%)	40 (55.56%)	34 (47.89%)	
Yes	69 (48.25%)	32 (44.44%)	37 (52.11%)	
**MPN subtype, nb (%)**				0.217
PMF	76 (53.15%)	38 (52.78%)	38 (53.52%)	
PV	32 (22.38%)	20 (27.78%)	12 (16.9%)	
ET	34 (23.78%)	14 (19.44%)	20 (28.17%)	
MDS/MPN	1 (0.70%)	0 (0%)	1 (1.41%)	
**Driver mutation, nb (%)**				0.083
JAK2	87 (60.84%)	51 (70.83%)	36 (50.70%)	
MPL	13 (9.09%)	4 (5.56%)	9 (12.68%)	
CALR	37 (25.87%)	14 (19.44%)	23 (32.39%)	
Triple negative	6 (4.20%)	3 (4.17%)	3 (4.23%)	
**IPSS at MF diagnosis, nb (%)**				**0.010**
Low or Intermediate-1	54 (37.76%)	16 (22.22%)	38 (53.52%)	
Intermediate-2 or High	52 (36.36%)	29 (40.28%)	23 (32.39%)	
Missing data	37 (25.87%)	27 (37.50%)	10 (14.08%)	
**DIPSS at molecular evaluation, nb (%)**				**0.024**
Low or Intermediate-1	86 (60.14%)	37 (51.39%)	49 (69.01%)	
Intermediate-2 or High	55 (38.46%)	35 (48.61%)	20 (28.17%)	
Missing data	2 (1.40%)	0 (0%)	2 (2.82%)	
**White Blood Count (G/L), median (IQR)**	9.9 (6.35; 16.60)	11.2 (6.97; 18.23)	9.625 (5.70; 15.50)	0.327
**Hemoglobin (g/dL), median (IQR)**	11.65 (9.90; 13.10)	11.25 (9.30; 12.80)	12 (10.55; 13.25)	**0.039**
**Circulating blasts, nb (%)**				0.560
No	66 (46.15%)	30 (41.67%)	36 (50.70)	
≥ 1%	43 (30.07%)	17 (23.61%)	26 (36.62%)	
Missing data	34 (23.78%)	25 (34.72%)	9 (12.68%)	
**Constitutional symptoms, nb (%)**				**0.001**
No	77 (53.85%)	25 (34.72%)	52 (73.24%)	
Yes	34 (23.78%)	23 (31.94%)	11 (15.49%)	
Missing data	32 (22.38%)	24 (33.33%)	8 (11.27%)	
**Karyotype, nb (%)**				0.354
Normal	55 (38.46%)	28 (38.89%)	27 (38.03%)	
Not normal excluding complex/monosomal	39 (27.27%)	20 (27.78%)	19 (26.76%)	
Complex/monosomal	3 (2.10%)	3 (4.17%)	0 (0%)	
Missing data	46 (32.17%)	21 (29.17%)	25 (35.21%)	
**HMR mutations, nb (%)**				**<0.001**
No	74 (51.75%)	26 (36.11%)	48 (67.61%)	
Yes	69 (48.25%)	46 (63.89%)	23 (32.39%)	

Table S2: Treatment type of non-ruxolitinib treated patients. *Nb=Number, ESA=Erythropoiesis Stimulating Agent, HSCT=Hematopoietic Stem Cell Transplantation.*

**Treatment type of non-ruxolitinib treated patients**	Nb of patients (n = 71)
No cytoreductive treatment	32
Hydroxyurea	16
Pegylated-interferon-alfa-2a (INF)	9
Hydroxyurea + INF	4
Revlimid	1
Thalidomid	1
Danatrol	1
EPO	2
Pipobroman	2
Anagrelide	1
Splenectomy	1
HSCT	1

Table S3: COX regression analysis of RAS pathway mutations acquisition associated factors, showing the impact of ruxolitinib treatment. *DIPSS= Dynamic International Prognostic Scoring System,HMR= high molecular risk mutations (ASXL1, EZH2, SRSF2, IDH1/2 and/or U2AF1). Two-sided COX proportional hazards regression was used for comparing the*

**Variables**	**Acquisition of RAS pathway mutations**		
	**HR**	**95% CI**	**pval**
**Ruxolitinib treatment**			
No	1	.	.
Yes	9.833	[1.226; 78.887]	**0.031**
**Age at molecular evaluation**	1.049	[0.988; 1.114]	0.121
**DIPSS at molecular evaluation**			
Low or Intermediate-1	1	.	.
Intermediate-2 or High	2.937	[0.784; 11.004]	0.110
**HMR mutations**			
No	1	.	.
Yes	3.094	[0.772; 12.392]	0.111

Table S4: Univariate and multivariate COX regression analysis of Overall Survival (OS) associated factors, showing the impact of RAS pathway mutations. *DIPSS= Dynamic International Prognostic Scoring System,HMR= high molecular risk mutations (ASXL1, EZH2, SRSF2, IDH1/2 and/or U2AF1). Two-sided COX proportional hazards regression was used for comparing the groups.*

**Variables**	**Univariate analysis**			**Multivariate analysis**		
	**HR**	**95% CI**	**pval**	**HR**	**95% CI**	**pval**
**RAS pathway mutation**						
No	1	.	.			
Yes	3.173	[1.573; 6.400]	**0.001**			0.0884
**Age at MF diagnosis**	1.071	[1.033; 1.111]	**<0.001**	1.064	[1.022; 1.107]	**0.002**
**DIPSS at molecular evaluation**						
Low or Intermediate-1	1	.	.	1	.	.
Intermediate-2 or High	3.236	[1.598; 6.551]	**0.001**	2.112	[1.032; 4.322]	**0.041**
**HMR mutations**						
No	1	.	.	1	.	.
Yes	2.717	[1.303; 5.662]	**0.008**	2.408	[1.152; 5.030]	**0.019**

Table S5: Univariate and multivariate COX regression analysis of AML/MDS transformation free survival (TFS) associated factors, showing the impact of RAS pathway mutations. *DIPSS= Dynamic International Prognostic Scoring System,HMR= high molecular risk mutations (ASXL1, EZH2, SRSF2, IDH1/2 and/or U2AF1). Two-sided COX proportional hazards regression was used for comparing the groups.*

Variables	Univariate analysis			Multivariate analysis		
	**HR**	**95% CI**	**pval**	**HR**	**95% CI**	**pval**
**RAS pathway mutation**						
No	1	.	.	1	.	.
Yes	5.167	[2.127; 12.549]	**<0.001**	3.020	[1.164; 7.831]	**0.023**
**Age at MF diagnosis**	1.095	[1.040; 1.153]	**0.001**	1.077	[1.017; 1.140]	**0.011**
**DIPSS at molecular evaluation**						
Low or Intermediate-1	1	.	.	1	.	.
Intermediate-2 or High	6.746	[2.231; 20.403]	**0.001**	3.656	[1.188; 11.248]	**0.024**
**HMR mutations**						
No	1	.	.	1	.	.
Yes	4.438	[1.482; 13.294]	**0.008**	5.572	[1.571; 19.760]	**0.008**

Table S6: Univariate and multivariate COX regression analysis of Overall Survival (OS) associated factors in ruxolitinib treated patients, showing the impact of RAS pathway mutations. *DIPSS= Dynamic International Prognostic Scoring System,MF=Myelofibrosis, HMR= high molecular risk mutations (ASXL1, EZH2, SRSF2, IDH1/2 and/or U2AF1). Two-sided COX proportional hazards regression was used for comparing the groups.*

**Variables**	**Univariate analysis**			**Multivariate analysis**		
	**HR**	**95% CI**	**pval**	**HR**	**95% CI**	**pval**
**RAS pathway mutation**						
No	1	.	.	1	.	.
Yes	4.084	[1.807; 9.231]	**0.001**	3.070	[1.337; 7.052]	**0.008**
**Age at MF diagnosis**	1.071	[1.024; 1.120]	**0.003**	1.067	[1.018; 1.118]	**0.007**
**DIPSS at molecular evaluation**						
Low or Intermediate-1	1	.	.			
Intermediate-2 or High	2.056	[0.907; 4.664]	0.084			
**HMR mutations**						
No	1	.	.	1	.	.
Yes	3.216	[1.200; 8.621]	**0.020**	3.400	[1.219; 9.484]	**0.019**

Table S7: Univariate and multivariate COX regression analysis of AML/MDS transformation free survival (TFS) associated factors in ruxolitinib treated patients, showing the impact of RAS pathway mutations. *DIPSS= Dynamic International Prognostic Scoring System, MF=Myelofibrosis, HMR= high molecular risk mutations (ASXL1, EZH2, SRSF2, IDH1/2 and/or U2AF1).* The Hazard Ratio for HMR mutations association with TFS could not be calculated due to the fact that none of the 26 ruxolitinib treated patients without HMR transformed into AML/MDS. Conversely, 13 patients among the 46 ruxolitinib treated patients harboring HMR mutations transformed to AML/MDS. Two-sided COX proportional hazards regression was used for comparing the groups.*

**Variables**	**Univariate analysis**			**Multivariate analysis**		
	**HR**	**95% CI**	**pval**	**HR**	**95% CI**	**pval**
**RAS pathway mutation**						
No	1	.	.	1	.	.
Yes	10.567	[3.105; 35.961]	**<0.001**	6.638	[1.910; 23.069]	**0.003**
**Age at MF diagnosis**	1.118	[1.035; 1.207]	**0.004**	1.089	[1.006; 1.178]	**0.034**
**DIPSS at molecular evaluation**						
Low or Intermediate-1	1	.	.	1	.	.
Intermediate-2 or High	6.602	[1.459; 29.877]	**0.014**			0.165
**HMR mutations***						
No	.	.	.			
Yes	.	.	.			

Table S8: Causes of death among non-ruxolitinib treated patients. *Nb=Number. MPN=Myeloproliferative Neoplasm. AML/MDS= Acute Myeloid Leukemia / Myelodysplastic syndrom.*

**Cause of death**	Nb of patients (n = 9/71)
Hemorrhage	1
Infection	3
Global condition deterioration	3
Unknown	2
**Disease status at death**	
Chronic MPN	5
AML/MDS	4

Table S9. List of probes and primers used for real time qPCR.

Targets	Conjugate	Reference/Sequence	Manufacturer
mJak2	FAM-MGB	Mm01208489_m1	Thermofisher
mGapdh	VIC-MGB	Mm99999915_g1	Thermofisher
hJAK2	FAM-MGB	Hs01078136_m1	Thermofisher
hNRAS	FAM-MGB	Hs00180035_m1	Thermofisher
hKRAS	FAM-MGB	Hs00364284_g1	Thermofisher
hASXL1	FAM-MGB	Hs00898215_g1	Thermofisher
hGAPDH	VIC-MGB	Hs02786624_g1	Thermofisher
mNras	None	Forward: ACGATCCAGCTAATCCAGAACCA	Sigma-Aldrich
		Reverse: GCAGGCAGGTCTCACCATCAAT	
mGapdh	None	Forward: CCTCGTCCCGTAGACAAAATGGT	Sigma-Aldrich
		Reverse: TGCAAATGGCAGCCCTGGTGAC	

Table S10. List of antibodies used for western blotting.

Target	Conjugate	Reference	Manufacturer
pSTAT3	No	9138S	Cell Signaling
STAT3	No	4904S	Cell Signaling
pSTAT5	No	9351S	Cell Signaling
STAT5	No	94205S	Cell Signaling
pERK	No	9101S	Cell Signaling
ERK	No	9102S	Cell Signaling
RAS	No	3965S	Cell Signaling
JAK2	No	3230S	Cell Signaling
ACTIN	No	MA5-11869	Invitrogen
Anti-mouse HRP	HRP	7076S	Cell Signaling
Anti-rabbit HRP	HRP	7074S	Cell Signaling

Table S11. List of shRNA sequences.

Designation	shRNA sequence
sh*Jak2* .1	TTCATTAAATATTAAATCTTCA
sh*Jak2* .2	TTAGCTATTCTCATCATGTCTA
sh*Jak2* .3	TTCAATGACATTTTCTCGCTCA
sh*Jak2* .4	TTTCATTAAATATTAAATCTTC
sh*Jak2* .5	TTTACATTGACTGAATTGCTGA
sh*Jak2* .6	TTACATTGACTGAATTGCTGAA
sh*Jak2* .7	TTTTACTTCAAACTGTTCTGTG
sh*Jak2* .8	AAATATTAAATCTTCATTCCTG
sh*Jak2* .9	TTTTACAAATTCTTGAACCAGA
sh*Jak2* .10	TTACTTTGTAGTATTCTTTGTC
shASXL1.1	TATTATATCAATGATATTCTGT
shASXL1.2	TAAAAATAAGAAACACACACTG

Table S12. List of genes and exons covered in our NGS panel.

Genes	Exons covered	NM used for variant calling
ASXL1	10, 12, 13	NM_015338
BRAF	15	NM_001374258
CALR	9	NM_004343
CBL	8,9	NM_005188
CCND2	All	NM_001759
CEBPA	All	NM_004364
CSF3R	All	NM_000760
CUX1	All	NM_181552
DNMT3A	All	NM_022552
ETV6	All	NM_001987
EZH2	All	NM_004456
FLT3	13, 14, 15, 20	NM_004119
HRAS	2, 3	NM_005343
IDH1	4	NM_005896
IDH2	4	NM_002168
IKZF1	All	NM_006060
JAK2	All	NM_004972
KIT	2, 8, 9, 10, 11, 13, 17, 18	NM_000222
KRAS	2, 3	NM_004985
MPL	All	NM_005373
NFE2	All	NM_001136023
NPM1	10, 11	NM_002520
NRAS	2, 3	NM_002524
PTPN11	3, 7, 8, 9, 10, 11, 12, 13	NM_002834
RUNX1	All	NM_001754
SETBP1	4	NM_015559
SF3B1	10, 11, 12, 13, 14, 15, 16	NM_012433
SH2B3	All	NM_005475
SRSF2	1	NM_001195427
TET2	All	NM_001127208
TP53	All	NM_000546
U2AF1	2, 3, 4, 5, 6	NM_006758
WT1	6, 7, 8, 9, 10	NM_024426
ZRSR2	All	NM_005089
