# Supplementary material for: JAK2 inhibition mediates clonal selection of RAS pathway mutations in myeloproliferative neoplasms
Source: Nat Commun. 2025 Jul 8;16:6270. doi: 10.1038/s41467-025-60884-1 (PMC12234676; doi:10.1038/s41467-025-60884-1)
Supplement: Supplementary file 2 — Description of Additional Supplementary Files [file 41467_2025_60884_MOESM2_ESM.pdf]

Description of Additional Supplementary Files

Supplementary Data 1: Myelofibrosis patients' molecular characteristics according to ruxolitinib treatment status. *RUXO= Ruxolitinib, NORUXO=Absence of Ruxolitinib treatment, VAF=Variant Allele Frequency. Only genes for which mutations are detected in at least one patient are presented.*

Supplementary Data 2: List of gene sets interrogated in the Gene Set Enrichment Analysis (GSEA) for JAK2 "low" versus JAK2 "high" patients within the Beat AML v2. dataset.
