# Supplementary material for: JAK2 inhibition mediates clonal selection of RAS pathway mutations in myeloproliferative neoplasms
Source: Nat Commun. 2025 Jul 8;16:6270. doi: 10.1038/s41467-025-60884-1 (PMC12234676; doi:10.1038/s41467-025-60884-1)
Supplement: Supplementary file 5 — Reporting Summary [file 41467_2025_60884_MOESM5_ESM.pdf]

nature portfolio

Corresponding author(s): Lina Benajiba

Last updated by author(s): Apr 14, 2025

Reporting Summary

Nature Portfolio wishes to improve the reproducibility of the work that we publish. This form provides structure for consistency and transparency in reporting. For further information on Nature Portfolio policies, see our Editorial Policies and the Editorial Policy Checklist.

Statistics

For all statistical analyses, confirm that the following items are present in the figure legend, table legend, main text, or Methods section.

n/a	Confirmed	
☐	☒	The exact sample size (*n*) for each experimental group/condition, given as a discrete number and unit of measurement
☐	☒	A statement on whether measurements were taken from distinct samples or whether the same sample was measured repeatedly
☐	☒	The statistical test(s) used AND whether they are one- or two-sided<br>*Only common tests should be described solely by name; describe more complex techniques in the Methods section.*
☐	☒	A description of all covariates tested
☐	☒	A description of any assumptions or corrections, such as tests of normality and adjustment for multiple comparisons
☐	☒	A full description of the statistical parameters including central tendency (e.g. means) or other basic estimates (e.g. regression coefficient) AND variation (e.g. standard deviation) or associated estimates of uncertainty (e.g. confidence intervals)
☐	☒	For null hypothesis testing, the test statistic (e.g. *F*, *t*, *r*) with confidence intervals, effect sizes, degrees of freedom and *P* value noted<br>*Give P values as exact values whenever suitable.*
☒	☐	For Bayesian analysis, information on the choice of priors and Markov chain Monte Carlo settings
☐	☒	For hierarchical and complex designs, identification of the appropriate level for tests and full reporting of outcomes
☒	☐	Estimates of effect sizes (e.g. Cohen's *d*, Pearson's *r*), indicating how they were calculated

Our web collection on statistics for biologists contains articles on many of the points above.

Software and code

Policy information about availability of computer code

Data collection	Flow cytometry data was collected using BD FACSDiva software Version 6.1.3 (BD Biosciences) on a Becton Dickinson FACSCanto II or LSRFortessa.<br>NGS sequencing was performed on a MiSeq instrument (Illumina).<br>scDNA sequencing was performed using NextSeq 550 instrument (Illumina).<br>Luminescence assays to determine cell viability were collected using a Spectramax i3x from Molecular Devices.<br>DNA/RNA concentration measurement: Thermo Fisher NanoDrop Lite.<br>Quantitative real-time PCR data was collected using QuantStudio 7 Flex with QuantStudio Software V1.1 (Applied Biosystems).<br>Blot image scanning with Cano Scan LiDE 300 (Canon).
Data analysis	All collected data were further analyzed with Microsoft Excel 2013, GraphPad Prism 8.0.1/2 and STATA software (STATA 15.1 Corporation, College Station, TX).<br>Figures were assembled using Adobe Illustrator and Photoshop CC2015.<br>The Gene Set enrichment analysis (GSEA) v4.2.0 software was used to identify functional associations of molecular phenotypes.<br>scDNAseq data were processed using the Tapestri Pipeline for cell calling, Genome Analysis Toolkit 4/haplotype caller for genotyping and the Tapestri Insights software. NGS data was analyzed using the SOPHIA DDM software. Cbioportal oncoprinter was used to generate oncoprint plots (https://www.cbioportal.org/oncoprinter). Fishplots were inferred generated using "fishplot" R package (version 4.1.1 for Mac).

For manuscripts utilizing custom algorithms or software that are central to the research but not yet described in published literature, software must be made available to editors and reviewers. We strongly encourage code deposition in a community repository (e.g. GitHub). See the Nature Portfolio guidelines for submitting code & software for further information.

Data

Policy information about availability of data

All manuscripts must include a data availability statement. This statement should provide the following information, where applicable:

- Accession codes, unique identifiers, or web links for publicly available datasets
- A description of any restrictions on data availability
- For clinical datasets or third party data, please ensure that the statement adheres to our policy

Human single cell DNA sequencing data generated in this study are available at the Sequence Read Archive (SRA) repository of the National Center for Biotechnology Information (NCBI). The accession number for these SRA data is PRJNA1222460 and data can be accessed through the following link: https://www.ncbi.nlm.nih.gov/sra/PRJNA1222460. Supplementary information, including Supplementary Figures and legends, Supplementary Tables, Supplementary Data and Source data are provided with this paper. Sources for reagents and cells are indicated in the Materials and Methods section. No custom code was generated in the course of this study.

Research involving human participants, their data, or biological material

Policy information about studies with human participants or human data. See also policy information about sex, gender (identity/presentation), and sexual orientation and race, ethnicity and racism.

Reporting on sex and gender	Sex of patients included in our retrospective study has been collected on medical charts and is reported in Table S1. The study was performed in accordance with the ethical guidelines of the Declaration of Helsinki, and was approved by our institutional review board (IRB00006477, CER-2020-55) including for sex collection.
Reporting on race, ethnicity, or other socially relevant groupings	No race, ethnicity or socially relevant data is reported in this study.
Population characteristics	Patient characteristics (age, sex, MPN sybtype, symptoms, blood counts, driver and additional somatic mutations, somatic karyotype, IPSS and DIPSS scoring systems) are reported in Table S1.
Recruitment	Recruitment of this retrospective study included all consecutive patients from whom a next generation sequencing (NGS) molecular analysis was performed at diagnosis and/or during follow-up, diagnosed with primary or secondary myelofibrosis (MF) according to WHO criteria and followed in Saint-Louis hospital between January 2011 and November 2019.
Ethics oversight	The study was performed in accordance with the ethical guidelines of the Declaration of Helsinki, and was approved by our institutional review board (IRB00006477, CER-2020-55).

Note that full information on the approval of the study protocol must also be provided in the manuscript.

Field-specific reporting

Please select the one below that is the best fit for your research. If you are not sure, read the appropriate sections before making your selection.

☒ Life sciences ☐ Behavioural & social sciences ☐ Ecological, evolutionary & environmental sciences

For a reference copy of the document with all sections, see nature.com/documents/nr-reporting-summary-flat.pdf

Life sciences study design

All studies must disclose on these points even when the disclosure is negative.

Sample size	Sample size calculations were not performed. As described in each figure legend, sample sizes ranged from n=2 to n=10 replicates per condition for in vitro studies and n=4 to n=5 mice per condition for in vivo studies. Sample sizes were sufficient to identify significant changes as indicated in each figure and for in vivo studies, sample size was chosen in light of the fact that these in vivo models were historically highly penetrant and consistent.
Data exclusions	No data were excluded from the analysis. Animals were excluded of the study if any signs of distress are observed without clinical signs of myeloproliferative neoplasm.
Replication	All attempts at replication were successful.
Randomization	All mice were randomized to treatment.
Blinding	No experiments were blinded as the reported data were not subjective but rather based on quantitative measures such as flow cytometry. Blinded observers visually inspected mice for obvious signs of illness such as loss of appetite, hunched posture, and lethargy.

Reporting for specific materials, systems and methods

We require information from authors about some types of materials, experimental systems and methods used in many studies. Here, indicate whether each material, system or method listed is relevant to your study. If you are not sure if a list item applies to your research, read the appropriate section before selecting a response.

Materials & experimental systems

n/a	Involved in the study
☐	☒ Antibodies
☐	☒ Eukaryotic cell lines
☒	☐ Palaeontology and archaeology
☐	☒ Animals and other organisms
☐	☒ Clinical data
☒	☐ Dual use research of concern
☒	☐ Plants

Methods

n/a	Involved in the study
☒	☐ ChIP-seq
☐	☒ Flow cytometry
☒	☐ MRI-based neuroimaging

Antibodies

Antibodies used	pSTAT3, STAT3, pSTAT5, STAT5, pERK, ERK, RAS, JAK2, ACTIN, Anti-mouse HRP, Anti-rabbit HRP, CD45.1, CD45.2. Antibody references (Catalog number and company) are detailed in the manuscripts' methods section.
Validation	All antibodies are commercially available and have been validated for the corresponding applications by the manufacturer. Antibody profiles and relevant citations are available on manufacturer's websites.

Eukaryotic cell lines

Policy information about cell lines and Sex and Gender in Research

Cell line source(s)	32D cell line was a kind gift of Dr Iannis Aifantis to Camille Lobry (Saint-Louis Research Institute, Paris, FRANCE). HEL cell line was purchased from the American Type Culture Collection (ATCC TIB180). UKE-1 cell line was a kind gift of Dr Walter Fiedler (University Hospital Eppendorf, Hamburg, GERMANY). Ba/F3 MPL-CALRWT and Ba/F3 MPL-CALRdel52 cell lines were a kind gift of Dr Isabelle Plo (Gustave Roussy Cancer Center, Villejuif, FRANCE).
Authentication	Identity of all cell lines was confirmed by short tandem repeat loci profiling.
Mycoplasma contamination	All cell lines were tested negative for Mycoplasma using MycoAlert Mycoplasma Detection Kit (Lonza #LT07–418), except Ba/F3 cell lines.
Commonly misidentified lines (See ICLAC register)	No cell lines were misclassified.

Animals and other research organisms

Policy information about studies involving animals; ARRIVE guidelines recommended for reporting animal research, and Sex and Gender in Research

Laboratory animals	Mus Musculus, C57BL/6JOlaHsd (Envigo), B6.SJL-PtprcaPepcb/BoyCrl Ly5.1 (Charles River Laboratories), hMRP8-NrasG12D mice (gift from Rose Ann Padua), Vav-cre (gift from Stéphane Giraudier), B6N.129S6(SJL)-Jak2tm1.1Ble/AmlyJ (Jackson labs), male or females, 6-12 weeks.
Wild animals	No wild animals were used in this study.
Reporting on sex	Male mice were used for one of our in vivo experiments, while female mice were used for the second one. Our results therefore apply to both males and females, in line with the fact that the studied disease is equally diagnosed in males and females.
Field-collected samples	No samples collected from the field were used in this study.
Ethics oversight	The French National Ethics Committee on Animal Care reviewed and approved all mouse experiments described in this study. Authorization number: APAFIS #34469-2021122222426491 v3. Housing conditions within Saint-Louis research institutes' animal facility satisfy The French National Ethics Committee on Animal Care requirements.

Note that full information on the approval of the study protocol must also be provided in the manuscript.

Clinical data

Policy information about clinical studies

All manuscripts should comply with the ICMJEguidelines for publication of clinical research and a completedCONSORT checklist must be included with all submissions.

Clinical trial registration	Not Applicable. Our clinical data report results from a retrospective study and not a clinical trial. The study was approved by our

	institutional review board (APHP Paris Nord IRB00006477, CER-2020-55).
Study protocol	Not Applicable
Data collection	Clinical and molecular characteristics were retrospectively collected from medical charts and electronic medical records.
Outcomes	Not Applicable

Plants

Seed stocks	Not Applicable
Novel plant genotypes	Not Applicable
Authentication	Not Applicable

Flow Cytometry

Plots

Confirm that:

- [x] The axis labels state the marker and fluorochrome used (e.g. CD4-FITC).
- [x] The axis scales are clearly visible. Include numbers along axes only for bottom left plot of group (a 'group' is an analysis of identical markers).
- [x] All plots are contour plots with outliers or pseudocolor plots.
- [x] A numerical value for number of cells or percentage (with statistics) is provided.

Methodology

Sample preparation	Sample preparations for flow cytometry are described in the "Methods" section.
Instrument	Data collection was performed on Becton Dickinson FACScanto II instrument.
Software	Becton Dickinson DIVA and FlowJo softwares. Data were further analysed with Microsoft Excel 2013 and GraphPad Prism 8.0.1.
Cell population abundance	The fraction of cells from each origin in our competition assays is reported in each figure and figure legend. The fraction of cells in each cell cycle phase is reported in the figures. The goal of our experiments was to compare by flow cytometry the proportion of each cell population in response to treatment exposure or after genetic remodeling.
Gating strategy	Live cells were gated on an FSC / SSC scatter plot.<br>Singlets were gated on an FSC-H / FSC-A scatter plot.<br>Proportion of GFP-positive, Crimson- positive, mCherry-positive, CD45.1/2-positive or PI-positive cells was then assessed on the singlet population.

- [x] Tick this box to confirm that a figure exemplifying the gating strategy is provided in the Supplementary Information.
